# Graph Feature Refinement and Fusion in Transformer for Structural Damage Detection

**DOI:** 10.3390/s24134415

**Published:** 2024-07-08

**Authors:** Tianjie Hu, Kejian Ma, Jianchun Xiao

**Affiliations:** 1 Research Center of Space Structures, Guizhou University, Guiyang 550025, China; gz_htj@hotmail.com (T.H.); makejian2002@163.com (K.M.); 2 Key Laboratory of Structural Engineering of Guizhou Province, Guiyang 550025, China

**Keywords:** structural damage detection, deep learning, CGsformer, graph convolutional network, global and local features, noise robustness

## Abstract

Structural damage detection is of significance for maintaining the structural health. Currently, data-driven deep learning approaches have emerged as a highly promising research field. However, little progress has been made in studying the relationship between the global and local information of structural response data. In this paper, we have presented an innovative Convolutional Enhancement and Graph Features Fusion in Transformer (CGsformer) network for structural damage detection. The proposed CGsformer network introduces an innovative approach for hierarchical learning from global to local information to extract acceleration response signal features for structural damage representation. The key advantage of this network is the integration of a graph convolutional network in the learning process, which enables the construction of a graph structure for global features. By incorporating node learning, the graph convolutional network filters out noise in the global features, thereby facilitating the extraction to more effective local features. In the verification based on the experimental data of four-story steel frame model experiment data and IASC-ASCE benchmark structure simulated data, the CGsformer network achieved damage identification accuracies of 92.44% and 96.71%, respectively. It surpassed the existing traditional damage detection methods based on deep learning. Notably, the model demonstrates good robustness under noisy conditions.

## 1. Introduction

As the foundational support system of buildings, the structure directly affects the building safety. Throughout the entire service life of buildings, structural damage is inevitable due to the coupling of adverse factors such as environmental corrosion, material aging, fatigue damage, sudden disasters, and the long-term effects of abnormal loads [1,2,3]. With the extension of service time, the load-bearing capacity and resistance to natural disasters of the structure decrease, thus leading to unforeseeable safety challenges for buildings [4,5,6]. Therefore, the early diagnosis and localization of structural damage play a vital role in the timely maintenance of structures [7]. Structural damage detection [8,9,10], as the core technology of Structural Health Monitoring (SHM), is essential for grasping the structural working status and evaluating structural safety [11]. Consequently, structural damage detection methods have emerged as a prominent research focus in the field of SHM [12,13]. Structural damage detection is categorized into local-based [14,15] and global-based [16,17] methods. Local-based methods are mainly exploited to detect small regular structures or the local structure, which can be challenging to assess damage from large and complex structures. To overcome the limitations of local-based methods, many global-based structural detection methods have been developed. Among them, vibration-based structural damage detection methods have garnered widespread attention due to their flexibility, efficiency, and wide application. Traditional vibration-based structural damage detection methods use natural frequency, modal shape, and other modal measurements to detect structural damage. Although these traditional methods exhibit excellent performance in specific application scenarios, they still have obvious limitations when confronting with complex recognition tasks.

With the rapid advancement of Machine Learning (ML) technology, ML provides new ideas for vibration-based structural damage detection methods and has been extensively applied [18,19,20,21]. Compared to traditional vibration-based methods, ML-based structural damage detection methods are able to automatically learn and identify damage characteristics from large amounts of collected data. These methods not only have the advantages of high efficiency, accuracy, and adaptability, but they also do not need to rely on the subjective experience of experts. Zhang et al. [22] put forward a SHM method based on incremental Support Vector Regression (SVR) and substructure strategy, which realized the health monitoring of large and complex structures. Hua et al. [23] proposed a method to compress features using Principal Component Analysis (PCA), which improved the classification accuracy of their SVR and reduced the computational cost. Salkhordeh et al. [24] designed a decision tree classifier based on the Bayesian optimization algorithm to select effective features for structural damage classification. Wang et al. [25] proposed a concrete corrosion prediction model, which applied eXtreme Gradient Boosting (XG-Boost) based on the Bayesian optimization algorithm to extract corrosion-related features, and they used a Random Forest (RF) model for prediction.

ML-based structural damage methods have achieved promising performance, but their key drawback is their reliance on predefined hand-crafted features. These hand-crafted features often contain elements irrelevant to the task, thus resulting in reduced model performance. Deep Learning (DL), as the most representative technology of machine learning, has demonstrated powerful capabilities in various data analysis fields. Many DL-related methods have been proposed [26,27] for vibration-based structural damage identification. These methods typically take acceleration response signals as input and then extract features through a predefined network, thus achieving better classification performance. In 2017, Abdeljaber et al. [28] proposed a structural damage detection algorithm based on nonparametric vibration and a Convolutional Neural Network (CNN), which extracted sensitive features from the original acceleration response signal to achieve vibration-based damage detection and location. Zhang et al. [29] trained a one-dimensional (1D) CNN to detect local structural stiffness and mass changes. In addition to a 1D CNN, Khodabandehlou et al. [30] introduced the 2D CNN, and the method employed multiple acceleration response signals collected by multiple sensors as the input of the 2D CNN. Tang et al. [31] divided an original time series and used a 2D CNN to extract the time–frequency image of the time series to obtain local features for structural anomaly detection. Mantawy et al. [32] encoded a time series into images and trained a 2D CNN to extract local information to describe structural health and damage status. Although the CNN has shown a powerful ability to represent local information of input signals for structural damage detection tasks, it has difficulty describing the long-term dependence of time series. To capture the global information of the data, Lin et al. [33] introduced the Long Short-Term Memory (LSTM) network for damage detection, which used the acceleration response signal as input to learn the signals’ long-term properties. Sony et al. [34] proposed an LSTM network to classify and locate structural damage by using structural acceleration response data. However, the local feature extraction capabilities of temporal modeling networks, the Recurrent Neural Network (RNN), and its variants are inefficient. To this end, subsequent work [35,36,37] has designed hybrid models, such as CNN and RNN (CNN-RNN), which combines the local feature extraction faculty of the CNN—with the ability to capture long-term dependencies—with the time series data of the RNN. Typically, these hybrid models integrate local and global features through parallel architectures [35,36] or learning strategies from local to global [38]. However, the former ignores the dependence between local and global features, and the latter leads to information loss and increases the complexity of the model. Therefore, how to effectively combine hybrid models to extract the local and global features of input data has become a challenging problem.

Although the aforementioned networks are able to extract local and global information from signals, they have difficulty in mining the intrinsic correlations of the data. Consequently, some researchers [39,40,41,42] have utilized irregular graphs to reflect the interconnections between structural damage data. Dang et al. [39] used the Graph Convolutional Network (GCN) to attain the intrinsic spatial coherence of sensor positions directly from structural vibration response data. Zhan et al. [41] exploited the GCN to construct graph structures in the wavelet domain for multisensor signals, and they trained the network with node classification as the goal for structural damage detection tasks. Wang et al. [40] proposed a waveguide-based dual GCN method for damage detection. The method employed Short-Time Fourier Transform (STFT) to obtain the spatial–temporal feature representation of the original waveguide signals. A local graph network was built using features obtained from samples, and then local graphs were formed by grouping nodes with similar types of damage, thus enabling damage detection on the structure. The successful applications of GCN-based methods show that the methods can effectively understand and represent the intrinsic correlations between original data and features.

There are still challenges in designing a deep learning method that accurately identifies structural damage. Firstly, both the CNN-based and RNN-based approaches have limitations in their abilities to model long-term dependencies and capture global temporal dependencies. Although RNNs are designed to deal with long-term dependencies, they may still encounter the problem of vanishing or exploding gradients when processing longer sequences. Secondly, some hybrid models usually use parallel networks, or they adopt the learning way from local to global, and there is a lack of investigation into the learning manner from global to local. Thirdly, in addition to the signal components related to the structural self-oscillation characteristics, the measured acceleration response signal inevitably contains noise. The direct input of such noisy data into deep learning models can affect the generalization ability and damage detection performance. Finally, research on structural damage methods based on graph convolution is limited.

According to the above analysis, we present a novel Convolutional Enhancement and Graph Features Fusion in Transformer Network (CGsformer) for detecting structural damage information. The proposed CGsformer network contains two contributions. On the one hand, the proposed network uses a hierarchical learning method from global to local to extract the characteristics of the acceleration response signal. Based on previous research work, global information can identify the periodic relationship of the acceleration vibration response signals, while local information can help to define and analyze the subtle differences in the acceleration response signals of short adjacent moments before and after damage occurs. Thus, a multihead self-attention was used to acquire the global information of the signals, and a convolution module was applied to represent the local information. On the other hand, the graph convolution module was embeded to enhance robustness against noise contamination. The graph convolution network constructs a graph structure for global features and filters out noise in global features through node learning, thus prompting the convolution module to extract more effective local features. On this basis, an extensive verification testing has been conducted by using the numerical model of the International Association for Structural Control (IASC)– American Society of Civil Engineers (ASCE) benchmark structure [43] and a four-story steel frame structure experiment. The study has compared the proposed CGsformer with deep learning-based models such as CNN, LSTM, and Transformer, especially in cases of limited datasets and noisy pollution scenarios.

## 2. Methodology

### 2.1. The Equation of Motion

Damage can cause detectable changes in the structural dynamic response. Therefore, it is usually possible to determine the structural health state by analyzing the structural response acceleration signals before and after damage. Without loss of generality, consider a linear multi-degree-of-freedom structure, whose motion equation is as follows [44]:

(1) $$M\ddot{u}\left(t\right)+C\dot{u}\left(t\right)+Ku\left(t\right)=F\left(t\right)$$

where M, C, and K represent the structural mass, damping, and stiffness matrices with the dimension of Z×Z, respectively. $u\left(t\right)$, $\dot{u}\left(t\right)$, and $\ddot{u}\left(t\right)$ denote the displacement, velocity, and acceleration with the dimension of Z×1, respectively; The superscript is a derivative of time. In addition, $F\left(t\right)$ represents the external loads with the dimension of Z×1.

It is generally believed that the damage will not lead to the change in the mass of the structure, but it will cause a decrease in the stiffness. Hence, the changes in stiffness of the structure before and after damage can be defined as ΔK

(2) $$\Delta K=K_{u}-K_{d}$$

This can be further expanded as the linear superposition of element stiffness matrices as follows:

(3) $$\Delta K=\sum_{i=1}^{n}a_{i}K_{u}^{i}$$

where $K_{u}^{i}$ defines the stiffness matrix of the *i*th element in the structure, and $a_{i}$ is the damage coefficient, which varies from 0 to 1. For example, $a_{i}=0.95$ means 5% stiffness lost in the *i*th element for stiffness.

The changes in stiffness can be reflected in the structural dynamic response. For example, damage can lead to a decrease in the structural vibration frequency and changes in the vibration modes. Therefore, collecting and analyzing structural responses can help us understand the health states of the structure. In practical applications, acceleration sensors, installed at different locations on the structure, are commonly used to capture these changes. The acceleration response refers to the measurement of the acceleration experienced by a structure under various loading conditions or external forces. This acceleration response data are usually collected by acceleration sensors.

This paper proposes a novel CGsformer network for structural damage detection. The proposed CGsformer network is not only able to extract global and local features in the acceleration response signal, but it also embeds the graph structure to better extract local features from global features. As far as we know, the proposed CGsformer unifies global features, local features, and graph structures for structural damage detection for the first time. At the same time, our experiments have proven that the proposed CGsformer has better classification performance and noise robustness.

### 2.2. Global and Local Feature Extraction

Research [35,36,45] has shown that the CNN-LSTM model has the ability to capture local and global features of signals, and its classification accuracy on structural damage detection tasks can surpass single CNN or RNN models. Inspired by previous studies, the multiheaded self-attention module and convolutional module were used to extract global and local features from structural damage detection signals.

**Figure 1:**
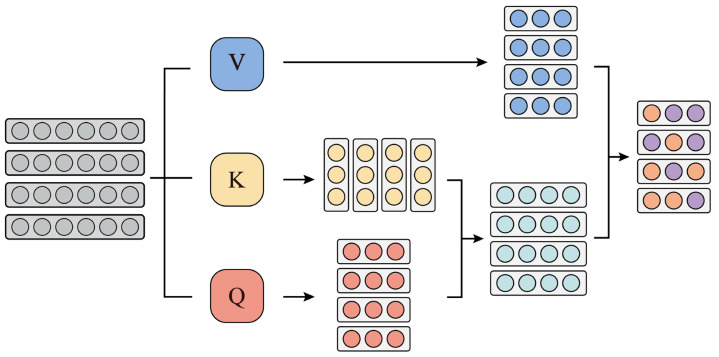
Self-attention mechanism.

**Multihead self-attention module:** This module comprises layer normalization, multihead self-attention, and dropout. Among them, multiheaded self-attention was proposed in [46,47]. To better understand multihead self-attention, we first describe self-attention. The self-attention mechanism aims to describe contextual information and capture the global characteristics of signals. The process of the self-attention mechanism is illustrated in Figure 1. Assume that matrix $X\in R^{M\times N}$ passes through linear matrices $W_{q}\in R^{M_{q}\times M}$, $W_{k}\in R^{M_{k}\times M}$, and $W_{v}\in R^{M_{v}\times M}$ to obtain query matrix $Q_{X}\in R^{M_{q}\times N}$, key matrix $K_{X}\in R^{M_{k}\times N}$, and value matrix $V_{X}\in R^{M_{v}\times N}$ as follows:

(4) $$\begin{array}{c} Q_{X}=W_{q}X\\ K_{X}=W_{k}X\\ V_{X}=W_{v}X \end{array}$$

**Figure 2:**
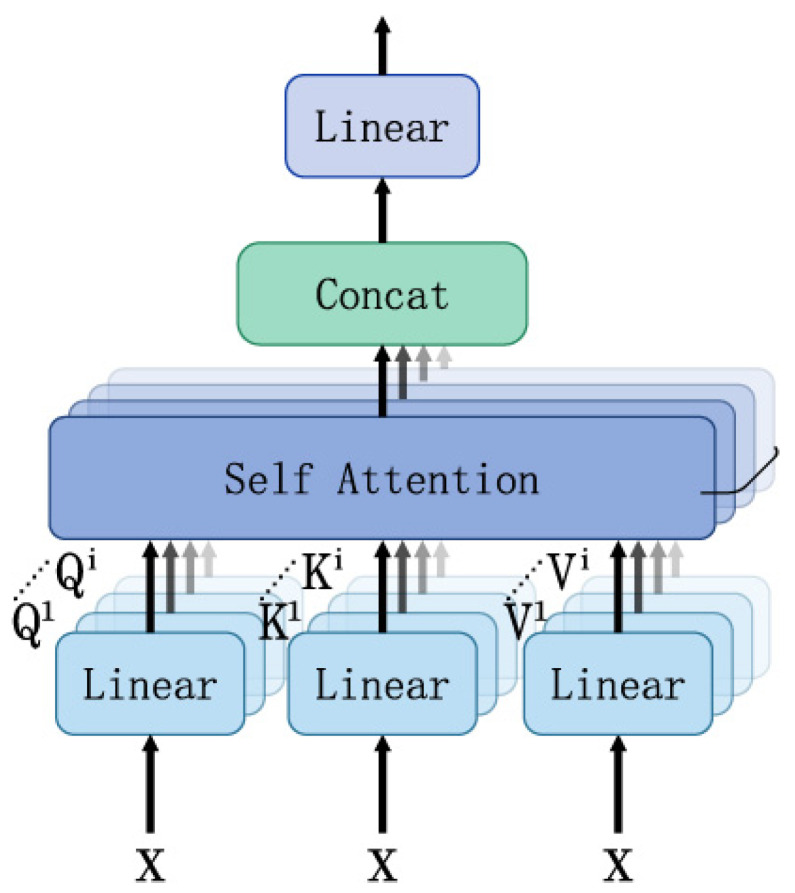
Multiheaded self-attention mechanism.

Subsequently, the attention score matrix $X_{score}$ is obtained by multiplying $Q_{X}$ and $K_{X}$ and by passing the softmax function. Finally, the attention coefficients matrix $X_{coff}$ is multiplied by the $V_{X}$ to acquire the attention coefficients. Similarly, the multiheaded self-attention mechanism uses multiple linear matrices $W_{q}^{i}$, $W_{k}^{i}$, and $W_{v}^{i}$ to obtain the matrices $Q^{i}$, $K^{i}$ and $V^{i}$, as shown in Figure 2. All attention coefficients are concatenated into multiheaded attention coefficients, which are obtained as follows:

(5) $$\begin{array}{c} M_{ac}\left(X\right)=Atten\left(W_{q}^{1}X,W_{k}^{1}X,W_{v}^{1}X\right)⊕\ldots \ldots \\ ⊕Atten\left(W_{q}^{i}X,W_{k}^{i}X,W_{v}^{i}X\right) \end{array}$$

where ⊕ represents the concatenate operation, and $Atten\left(\cdot \right)$ denotes the self-attention operations.

**Figure 3:**
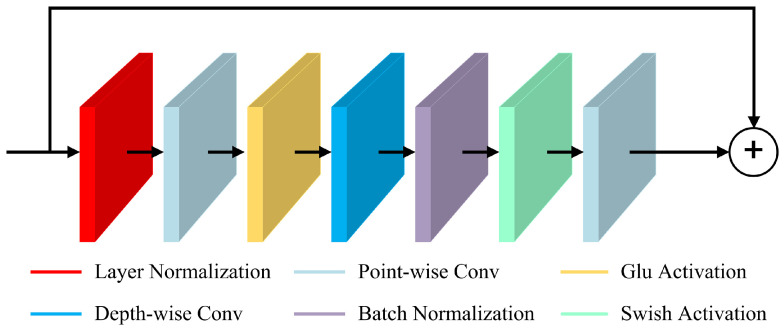
The convolution module.

**Convolution module:** The convolution module includes a pointwise convolution operation with 1x1 convolution kernel, and it doubles the number of channels. Next, the GLU activation function is utilized to control the number of output features. Batch normalization is carried out to stabilize the internal distribution of the network after applying a depthwise convolution. Finally, a Swish activation function and pointwise convolution are employed to complete the processing of the whole module. The convolution module is depicted in Figure 3.

### 2.3. Graph Convolution Network

As a data structure, a graph can effectively describe the association between two nodes, which is represented as follows [48],

(6) $$G=\left(G_{nodes},\;G_{edges}\right)$$

where $G_{nodes}$ indicates the set of nodes, and $G_{edges}$ represents the set of edges. The Graph Neural Network (GNN) is a deep learning model designed for processing graph-structured data, which are widely used in fields such as social network analysis, molecular structure modeling, and recommendation systems. The GCN is an important variant of GNNs, which is able to construct a graph structure from the input sequence or features and learn the adjacency relationships between nodes to enhance the understanding and representation of global information.

We assume that the input sequence data features are $X\in R^{n\times d}$. To construct a graph for X, we first need to create nodes from the sequence and then define edges based on these created nodes. Each element in the sequence is considered as a node in the graph network. After node construction, the creation of edges is done through a self-attention mechanism on the nodes. When we have completed the construction of the nodes and edges, a graph is formed that contains rich and reliable connections between relevant nodes. Specifically, an input X can be represented by a GCN layer as follows:

(7) $$X^{\left(l+1\right)}=M_{Lap}X^{\left(l\right)}W^{\left(l\right)}$$

where $X^{\left(l\right)}$ and $W^{\left(l\right)}$ are the input and learnable matrix for the *l*th layer, respectively, and $M_{Lap}$ is the Laplacian matrix used to represent the topological structure of the graph, which is denoted as

(8) $$M_{Lap}=D^{-\frac{1}{2}}\hat{A}D^{-\frac{1}{2}}$$

where $D=\mathrm{diag}\left(d_{1},\;d_{2},\;\ldots ,\;d_{n}\right)$ is the degree matrix used to describe the number of edges $d_{n}$ corresponding to node n, and $\hat{A}$ is the adjacency matrix used to represent the relationships between nodes, which is defined as

(9) $$\hat{A}=\;M_{re}\left(\frac{{\left(W_{d}X\right)}^{T}\left(W_{n}X\right)}{d}\right)\in R^{n\times n}$$

where $W_{d}$ and $W_{n}$ denote the learnable matrices, *d* represents the feature dimension of the whole sequence, *T* denotes the transpose operation, and $M_{re}\left(\cdot \right)$ represents ReLU activation function, which is utilized as the activation function filters out negative links between nodes. Negative links imply that there is no necessary direct connection between these two nodes. Overall, the GCN enhances the correlation between local features and suppresses noise through graph structure, thus achieving better global modeling of features.

### 2.4. Extracting Robust Features via CGsformer

Traditional hybrid models usually adopt parallel architecture or learn from local to global methods. However, the former assumes that global information and local information are independent, while the latter will lead to the loss of some global information and increase the complexity of the model. To this end, we proposed the CGsformer network, which adopts a learning manner from global to local.

**Figure 4:**
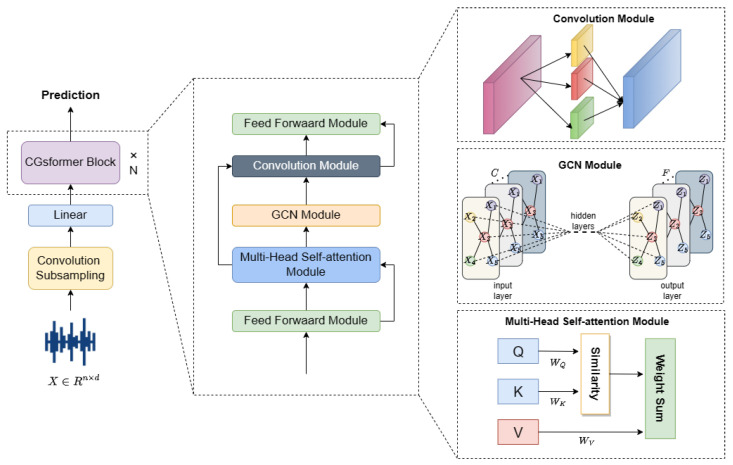
The overall structure of the CGsformer. The proposed CGsformer is mainly composed of four parts: feedforward module, self-attention mechanism module, convolution module, and graph network module.

Figure 4 illustrates the proposed CGsformer network. The innovation and advantage of the proposed CGsformer network lies in the hierarchical learning from global to local to extract acceleration response signal features for representing structural damage. Meanwhile, embedding GCN into the learning process of global to local features aims to build a graph structure for global features. By learning node features of global information to filter out noise and retain important global features, the convolution module learns more effective local features.

**Figure 5:**
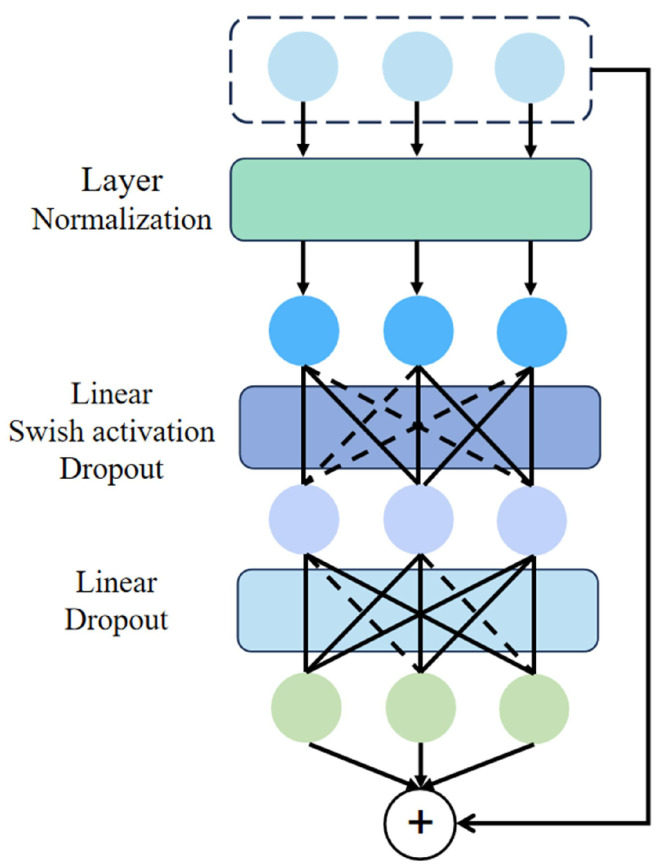
The feedforward module.

The shallow features of the acceleration response signal are obtained through convolutional subsampling and linear layers, and they are input into the CGsformer block to extract deep global and local features. In the CGsformer block, the feedforward module, as shown in Figure 5, is first used to achieve independent mapping of each position in the sequence. The feedforward module consists of two linear projections and an intermediate nonlinear activation function, where the first linear layer extends the feature dimensionality of the data by four times, and the other linear layer projects it to the original model dimension. We normalize the network using the Swish activation function, dropout, and layer normalization operation in the feedforward module. The Swish function is used to nonlinearly transform the input signal, and dropout randomly discards neurons to prevent the network from overfitting. The layer normalization operation speeds up network training and convergence. Simultaneously, the entire module follows the prenormalized residual unit.

Then, the multiheaded self-attention module realizes the long-term dependency modeling of the structural acceleration response data so that the weights of each position can be dynamically adjusted according to the contextual information of different positions. The graph convolution module captures the connection relationship between different nodes to better understand the local and global dependencies in the structural acceleration response data from the perspective of graph structure. The convolution module completes the further capture of local features of the structural acceleration response data.

According to the above description, the process of the CGsformer model is unfolded as follows:

(10) $$\begin{array}{c} \tilde{X}\;=\;X\;+\;\frac{1}{2}M_{ff}\left(X\right)\\ \hat{X}\;=\;\tilde{X}\;+\;M_{ac}\left(\tilde{X}\right)\\ \hat{X}\;=\;M_{gcn}\left(\hat{X}\right)\\ \bar{X}\;=\;\hat{X}\;+\;M_{conv}\left(\hat{X}\right)\\ Y\;=\;M_{norm}\left(\;\bar{X}\;+\;\frac{1}{2}M_{ff}\left(\bar{X}\right)\right) \end{array}$$

where $M_{ff}\left(\cdot \right)$ defines the feedforward module, $M_{ac}\left(\cdot \right)$ is the multiheaded self-attention module, $M_{gcn}\left(\cdot \right)$ is the graph convolution module, $M_{conv}\left(\cdot \right)$ is the convolution module, $M_{norm}\left(\cdot \right)$ is the layer normalization operation, and $Y\in R^{n\times d}$ denotes the prediction result of the damage patterns after CGsformer output.

### 2.5. Prediction

Finally, we pass the features *Y* obtained from the CGsformer model through two Fully Connected (FC) layers to obtain the final damage category. The process is as follows:

(11) $$M_{pre}\;=\;W_{2}\left(M_{re}\left(W_{1}Y\;+\;b_{1}\right)\right)\;+\;b_{2}\in R^{d_{out}}$$

where $W_{1}$ and $W_{2}$ represent weights of the FC layers, $b_{1}$ and $b_{2}$ represent biases of the FC layers, and $d_{out}$ represents the dimension of the output classes.

## 3. Verification by Simulation

In this section, we validated the damage detection performance of the CGsformer model through the phase I IASC-ASCE SHM numerical benchmark structure [43].

### 3.1. Test Setup and Data Preparation

**Figure 6:**
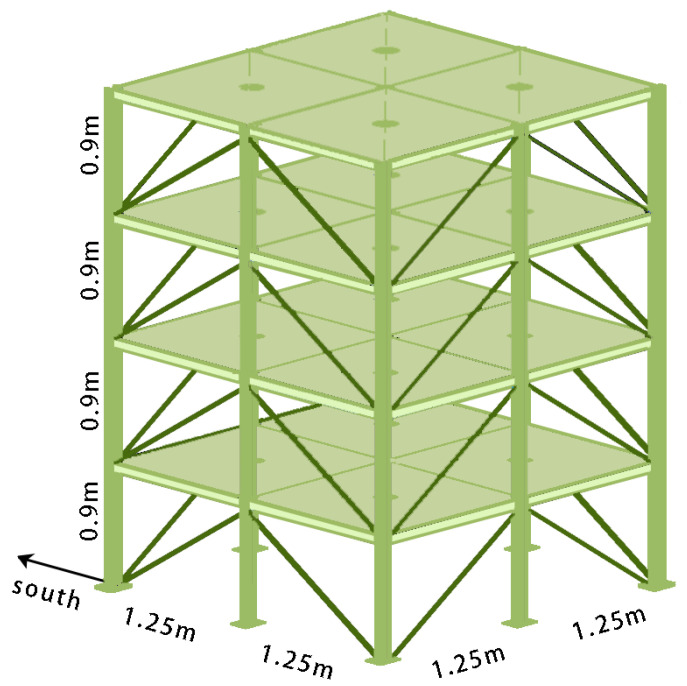
IASC-ASCE SHM Benchmark structure model.

**IASC-ASCE SHM benchmark dataset:** The numerical model of the IASC-ASCE SHM benchmark has been jointly established by the IASC and the ASCE [43], which provides a standardized and unified benchmarking platform for comparing and evaluating various structural health monitoring methods. As shown in Figure 6, the IASC-ASCE SHM benchmark structure is a $\frac{1}{3}$-scale steel frame model with four stories. The model has a story height of 0.9 m and a total height of 3.6 m. The plan size of the model is 2.5 m × 2.5 m, with each bay spanning 1.25 m. Each story consists of nine steel columns and eight diagonal braces. Each floor slab is composed of four uniformly distributed mass plates. The first floor has four 800 kg plates. The second and third floors each have four 600 kg plates. The fourth floor has three 400 kg plates and one 550 kg plate, which are arranged to give the structure an asymmetric mass distribution. The structure is excited by an external excitation acting in the diagonal direction at the top. For more detailed information on the IASC-ASCE SHM simulated benchmark structure, please consult reference [43].

**Figure 7:**
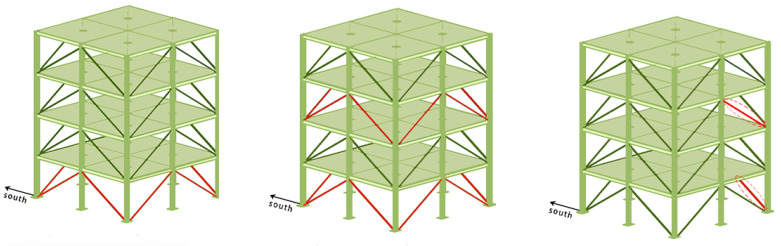
From left to right, they correspond to damage modes D.P.1, D.P.2, and D.P.4.

The model defines a total of six damage patterns. Figure 7 illustrates three of these damage patterns: D.P.1, D.P.2, and D.P.4. This paper focuses on these damage patterns, and the original acceleration vibration response data were generated using the 12-degree-of-freedom finite element model in the MATLAB program from the IASC-ASCE SHM Research Group. The acceleration response data for these four damage modes (D.P.0, D.P.1, D.P.2, and D.P.4) were collected from four sensors on each floor, as shown in Table 1. The noise levels used were 0%, 20%, and 50%, respectively. The data were preprocessed, thus resulting in training (validation and testing) data for the classifiers. The dataset among the 24 contains a total of 6248 samples, with 4000 samples used for training, 1000 used samples for validation, and 1248 used samples for testing. The preprocessing procedure is described in detail in the next step.

Using Gaussian white noise to simulate environmental excitation is a common method. Gaussian white noise is a random process with a uniform power spectral density, which is characterized by equal and independent power components at all frequencies. The power spectral density of white noise is constant across all frequencies.

(12) $$S\left(f\right)\;=\;\frac{N_{0}}{2}$$

where $N_{0}$ is the intensity of the noise power spectral density.

**Figure 8:**
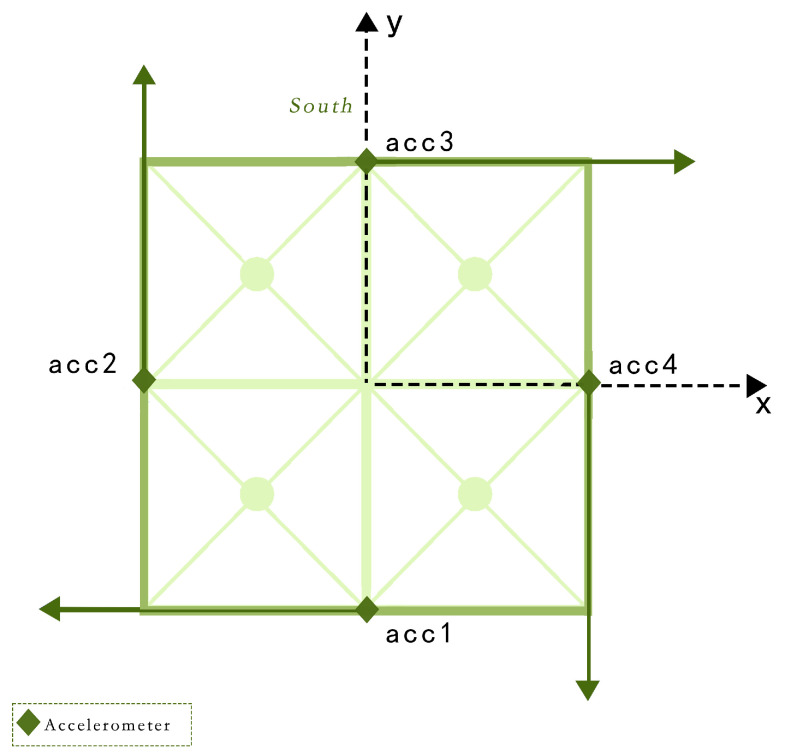
Distribution of IASC-ASCE SHM Benchmark model measurement points.

**Data preprocessing:** First, the response data collected on two acceleration sensors in the same direction on each floor were fused to obtain the fused translation data of each floor from the *x* and *y* directions [49] as follows:

(13) $$\left\{\begin{array}{c} {acc}_{x,d}\;=\;0.5\times \left({acc}_{1,d}\;+\;{acc}_{3,d}\right)\\ {acc}_{y,d}\;=\;0.5\times \left({acc}_{2,d}\;+\;{acc}_{4,d}\right) \end{array}\right.$$

where $acc_{1,d}$, $acc_{2,d}$, $acc_{3,d}$, and $acc_{4,d}$ denote the acceleration time history response data collected by the four sensors in the IASC-ASCE SHM benchmark structural model for floor d, as depicted in Figure 8, respectively, and $acc_{x,d}$ and $acc_{y,d}$ denote the translational acceleration in the *x* and *y* directions of the *d* floor, all of which are 1D temporal data.

Next, the sampling points of the sensor were calculated. Assume that the sampling rate of the sensors is $f_{s}$, and the sampling time is $t_{s}$ when collecting the original acceleration time history response data of the four sensors in floor *d*. Correspondingly, the number of sampling points $S_{j}$ for one sensor in sampling time $t_{s}$ is $S_{j}=f_{s}\times t_{s}$. The sampling points $S_{j}$ are divided into *m* nonoverlapping data segments with fixed lengths of 128. The translational acceleration datasets for completing the segmentation process in floor *d* are represented as $acc_{x,d}^{\prime }$ and $acc_{y,d}^{\prime }$. Meanwhile, $acc_{x,d}^{\prime }$ and $acc_{y,d}^{\prime }$ are normalized and shuffled, and the processed data are defined as $acc_{x,d}^{\prime \prime }$, and $acc_{y,d}^{\prime \prime }$. According to the above method, the data from each sensor on every floor under other damage modes are processed, thus resulting in a dataset for each direction of every floor under each working condition. Assuming that the damage detection task contains a total of *p* damage patterns, the translational acceleration dataset *D* for all floors in the *x* and *y* directions can be expressed as

(14) $$\begin{array}{c} D=[[{acc}_{x,1,1}^{\prime \prime }{acc}_{y,1,1}^{\prime \prime }\ldots {acc}_{x,d,1}^{\prime \prime }{acc}_{y,d,1}^{\prime \prime }\ldots {acc}_{x,4,1}^{\prime \prime }{acc}_{y,4,1}^{\prime \prime }],\\ \;[{acc}_{x,1,2}^{\prime \prime }{acc}_{y,1,2}^{\prime \prime }\ldots {acc}_{x,d,2}^{\prime \prime }{acc}_{y,d,2}^{\prime \prime }\ldots {acc}_{x,4,2}^{\prime \prime }{acc}_{y,4,2}^{\prime \prime }],\\ \;\ldots \;\\ \;[{acc}_{x,1,p}^{\prime \prime }{acc}_{y,1,p}^{\prime \prime }\ldots {acc}_{x,d,p}^{\prime \prime }{acc}_{y,d,p}^{\prime \prime }\ldots {acc}_{x,4,p}^{\prime \prime }{acc}_{y,4,p}^{\prime \prime }]]. \end{array}$$

The translational acceleration dataset *D* is then divided into columns to obtain eight data subsets. Each subset represents the acceleration response data of a certain translational direction in a certain floor, and contain *p* damage patterns.

Therefore, the proposed CGsformer with first floor *x* direction acceleration time history response data will be introduced, i.e., $D_{x,1,p}=\;\left[{acc}_{x,1,1}^{\prime \prime }\;{acc}_{x,1,2}^{\prime \prime }\;\ldots \;{acc}_{x,1,p}^{\prime \prime }\right]$ containing *p* damage patterns. More generally, we define the $D_{x,1,p}$ as $X\in R^{n\times d}$.

**Implementation of details:** The hyperparameters of the CGsformer are set as shown in Table 2. All hyperparameters were determined by performing ablation experiments on damaged structures in the first floor in the x direction. And, the same hyperparameter settings were used for all experiments. The entire model used the Adam optimizer for gradient descent, and the learning rate was 0.001. The loss was computed using the crossentropy function. Training and testing were performed on a machine with a Tesla A100 GPU using a batch size of 32.

### 3.2. Comparison with Other Models

**Figure 9:**
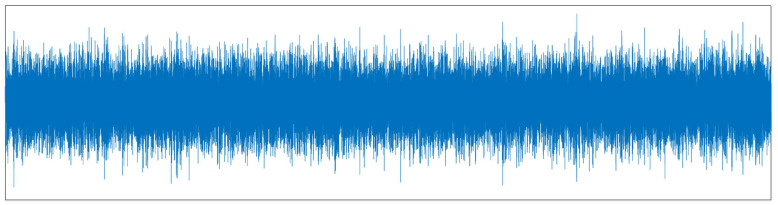
Acceleration time response curve of D.P.1 damage pattern, which was collected from sensor NO.1 (acc 1) on the first floor at 0 noise level.

**Figure 10:**
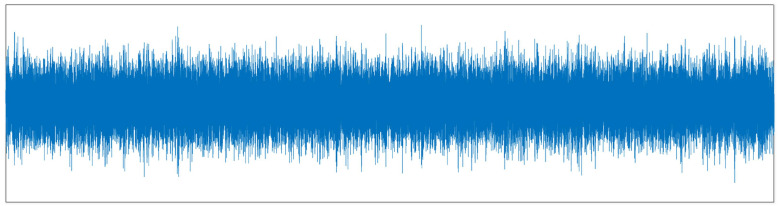
Acceleration time response curve of D.P.1 damage pattern, which was collected from sensor NO.3 (acc 3) on the first floor at 0 noise level.

To verify that the accuracy performance of CGsformer is better than other models, the performance of the proposed CGsformer structural damage detection method was compared with other methods on the IASC-ASCE SHM benchmark structural dataset with an initial layer *x* direction noise level of 0%, and the related acceleration response curves are illustrated in Figure 9 and Figure 10. The comparative models include following:

- **CNN** [28]: In this experiment, a one-dimension (1D) convolution operation with two convolutional kernels of size five constructed the network.
- **LSTM** [33]: In this experiment, a bidirectional LSTM with two hidden layers and a dimension of 128 constructed the network.
- **CNN-LSTM** [37]: The spatial features were first extracted using a 1D CNN with a convolutional kernel size of 15, and then these features were input into a two-layer LSTM with a hidden layer dimension of 256 for temporal modeling.
- **Multihead CNN** [50]: Multihead CNN learns different-scale or different-type features by introducing multiple parallel convolutional branches. Each branch can focus on different spatial or frequency domain information, and their results are fused to more comprehensively describe structural damage information.
- **Transformer** [46]: In this experiment, four Transformer blocks were used with eight heads in the multiheaded attention mechanism, and the dimension was set to 512.
- **Conformer** [51]: Conformer combines the advantages of CNN and self-attention mechanisms, effectively handles long input sequences, and possesses strong modeling and contextual understanding capabilities. The experimental hyperparameter settings for Conformer were consistent with the CGsformer, as illustrated in Table 2.

To measure the classification performance of the different models, the accuracy performance ***ACC*** and F1 score ($F_{score}$) were adopted as indicators. The accuracy performance ACC and F1 score are defined as follows

(15) $$\mathrm{ACC}=\frac{X_{TP}+X_{TN}}{X_{TP}+X_{TN}+X_{FP}+X_{FN}}$$

(16) $$F_{\mathrm{score}}=\frac{2X_{pre}X_{rec}}{X_{pre}+X_{rec}}$$

where $X_{TP}$, $X_{TN}$, $X_{FP}$, and $X_{FN}$ represent the true positive, true negative, false positive, and false negative values of data samples, respectively; $X_{pre}$ and $X_{rec}$ are the precision and recall rates, respectively, which are denoted as follows:

(17) $$X_{pre}=\frac{X_{TP}}{X_{TP}+T_{FP}}$$

(18) $$X_{rec}=\frac{X_{TP}}{X_{TP}+T_{FN}}$$

Table 3 presents the classification performance of the different models on the dataset. The proposed CGsformer achieved the best performance. The single CNN and LSTM models only considered the local information or global information of the input signals, thus resulting in relatively poor results. Compared to the CNN, the multihead CNN could learn features of different scales, and its accuracy was 3.45% higher than the CNN, thus reaching 92.39%. However, the multihead CNN still failed to capture the long-term information of the signals. The CNN-LSTM model took into account both local information and long-term dependencies to better capture the characteristics of the structural damage, with an accuracy of 92.55%. Compared to the CNN-LSTM model, the Transformer model captured more robust global dependencies through the self-attention mechanism and achieved a gain of 1.52%. The Conformer model employed the advantages of the CNN in capturing local features and the Transformer in capturing global features, with an accuracy of 95.27%. The proposed CGsformer model embedded graph structures into both global and local features. This not only effectively filtered out noise interference from global features, but it also helped the convolution module better understand global information, thereby extracting more effective local features. In Table 3, the identification accuracy of the proposed CGsformer is shown at 96.71%, thus achieving the best classification performance.

In order to test whether the proposed CGsformer model is significantly relevant to other models, the 95% confidence interval (CI) was utilized to test the statistical significance of the accuracy performance between the proposed model and the other models. In Table 4, we present the accuracy differences (ΔACC), 95% confidence intervals (CIs), and confidence intervals for the accuracy differences between the proposed model and other models (ΔCI), where $\Delta \mathrm{ACC}={\mathrm{ACC}}_{a}-{\mathrm{ACC}}_{b}$ represents the difference in accuracy between the proposed model ${\mathrm{ACC}}_{a}$ and other models ${\mathrm{ACC}}_{b}$—the 95% CI—which is denoted as

(19) $$CI=ACC\pm z\cdot SE,SE=\sqrt{\frac{ACC\left(1-ACC\right)}{n_{t}}}$$

where *z* was set to 1.96 at the 95% CI, $n_{t}$ is equivalent to the size of test samples, and ΔCI is formulated as follows:

(20) $$\Delta \mathrm{CI}=\Delta \mathrm{ACC}\pm z\cdot \sqrt{SE_{a}^{2}+SE_{b}^{2}}$$

It can be intuitively seen from Table 4 that the proposed CGsformer model had the most accurate predictive performance. Moreover, the CI of the proposed model was the narrowest, which indicates that the proposed model is more robust. Meanwhile, the proposed model had significant statistical significance compared to other models (except for Conformer). In conclusion, the proposed model is superior to the other models.

### 3.3. Ablation Study

**Figure 11:**
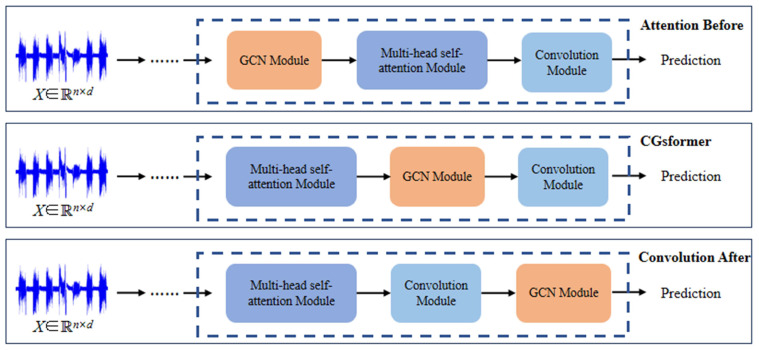
Illustration of ablation experiments with GCN placed at different positions in the CGsformer block.

In this subsection, the performance of the GCN module is compared at different positions in the model, and more conclusions are drawn. As shown in Figure 11, three combinations were set. The graph convolution module was placed before the multihead self-attention module, after the convolution module, and in between the two for the proposed CGsformer model. They are named Attention Before, Conversion After, and CGsformer, respectively.

The accuracy performance outcomes are presented in Table 5, and it can be found that the results of the three combinations were better than that of Conformer. Among the three combinations, the proposed CGsformer model achieved the best performance, followed by Attention Before and the relatively poor Convolution After. Based on these observations, the following conclusions can be drawn: (1) All three models outperformed the Conformer model: this means that graph structure learning can help the model select more discriminative features. (2) Placing the GCN module before the multihead self-attention module failed to capture the higher-order information of the current sequence features and only relied on shallow features for sequence representation learning, thus resulting in poorer performance. Placing the GCN module after the convolution module aggregated the final decision features, but this operation failed to fully utilize the semantic information represented by attention and loses effectiveness. (3) The proposed CGsformer model achieved the best performance, with an accuracy of 96.71%. Graph convolution learning of features after the self-attention module can further propagate global features to adjacent nodes, which helps to better understand and express node features by combining local adjacency information.

### 3.4. Comparative Analysis on the Four-Story Numerical Model with 24 Classifiers

As mentioned earlier, the acceleration response data from all measurement points of the IASC-ASCE SHM benchmark structure collected under four damage patterns were used to validate the proposed CGsformer-based damage detection method in this paper. The acceleration sensors at all measurement points on each floor had a sampling frequency of $f_{s}=250$ Hz for all damage patterns, and the vibration response was measured over a duration of 800 s. Therefore, the number of sampling points $S_{j}$ for the sensor within the sampling time was 200,000.

A total of 24 CGsformer classifiers (3 noise levels × 4 floors × 2 translational directions) were trained using the acceleration response data collected from all acceleration sensors of the IASC-ASCE SHM benchmark structure under three different noise levels (0%, 20%, and 50%). The collected data were tackled with the steps outlined in Section 3.1. Therefore, the dataset of the acceleration time history response data for each direction of every floor under each noise level contains 6248 samples (4 damage patterns × 1562 data segments). Among these, 4000 samples were used for training the classifiers, 1000 samples for validation, and 1248 samples for testing the trained classifiers.

**Figure 12:**
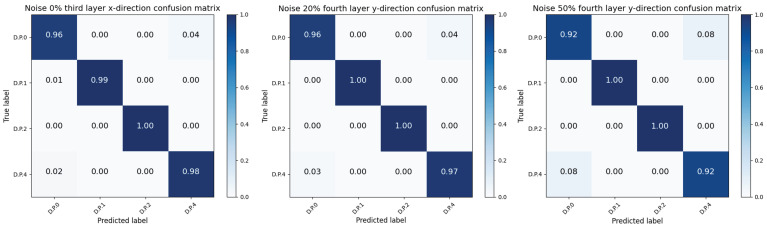
Diagram of the best-performing confusion matrix for the three noise levels.

The ***ACC*** results are reported in Table 6, and some observations can be made: (1) At different noise levels, increasing noise levels led to a decrease in accuracy. The highest average accuracy of 97.25% was achieved at a noise level of 0% (with the highest detection accuracy classifier appearing in the third floor, *y* direction, with an accuracy of 98.24%). At a noise level of 20%, the average accuracy reached 96.74% (with the highest detection accuracy classifier appearing in the fourth floor, *y* direction, with an accuracy of 98.32%). At a noise level of 50%, the average accuracy was 95.44% (with the highest detection accuracy classifier also appearing in the fourth floor, *y* direction, with an accuracy of 96.15%). The confusion matrix with the highest accuracy for the three noise levels is shown in Figure 12. As can be seen from Figure 12, misclassifications primarily occurred between D.P.0 and D.P.4 at all three noise levels, with the error rate increasing as the noise levels rose. This result is attributed to the training samples being insufficiently diverse and the model’s limited sensitivity to the highly similar overlapping features in their acceleration response signals. Although increasing noise levels can lead to decreased accuracy, comparing the damage detection results between 0% and 50% noise levels shows that the average detection accuracy of the four-floor, eight-classifier model only decreased by 1.81%. This indicates that the proposed CGsformer-based damage detection model has strong noise resistance. (2) The CGsformer model exhibited stronger robustness as the noise increased. Taking the first floor, *x* direction, as an example, even with an additional 20% noise data, the detection accuracy reached 96.07%. When the noise levels were 0%, 20%, and 50%, the average accuracy values of the Conformer model were 96.18%, 95.25%, and 94.58%, respectively. Comparing the results of the CGsformer model in Table 6, the CGsformer model achieved better results, with an improvement of 1.07%, 1.49%, and 0.86% for the noise levels at 0%, 20%, and 50%, respectively. (3) Despite the suboptimal performance compared to low noise levels, the model still achieved results comparable to the multihead CNN model (92.39%) when dealing with a noise level of 50%. This indicates that the CGsformer model has further learned the correlation between local and global features, thus exhibiting stronger generalization even when half of the data is noisy.

## 4. Experimental Verfication

In this section, the performance of the proposed CGsformer model is further compared with other models in a four-story, single-span, steel frame structure to verify the effectiveness of the proposed CGsformer model in different structures through testing.

### 4.1. Experiment Description

**Figure 13:**
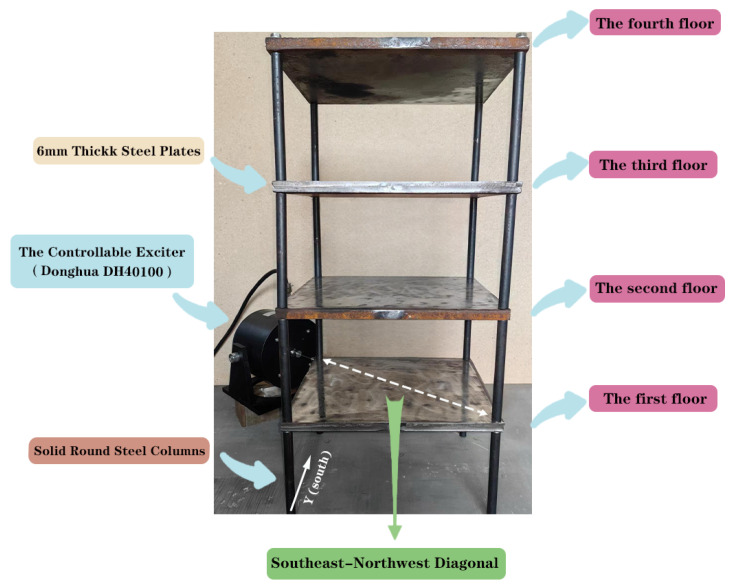
The four-story steel frame structure experimental model.

As depicted in Figure 13, the experimental structure is a four-story, single-span, steel frame structure. The plan dimension of the structure is 260 mm × 320 mm, with a total height of 672 mm. All floor slabs have been constructed from 16 mm thick steel plates and are supported by four solid round steel columns. Each column has a height of 152 mm and a cross-section diameter of 16 mm. The structural members are made from grade Q235 steel, which has a nominal yield stress of 235 MPa.

**Figure 14:**
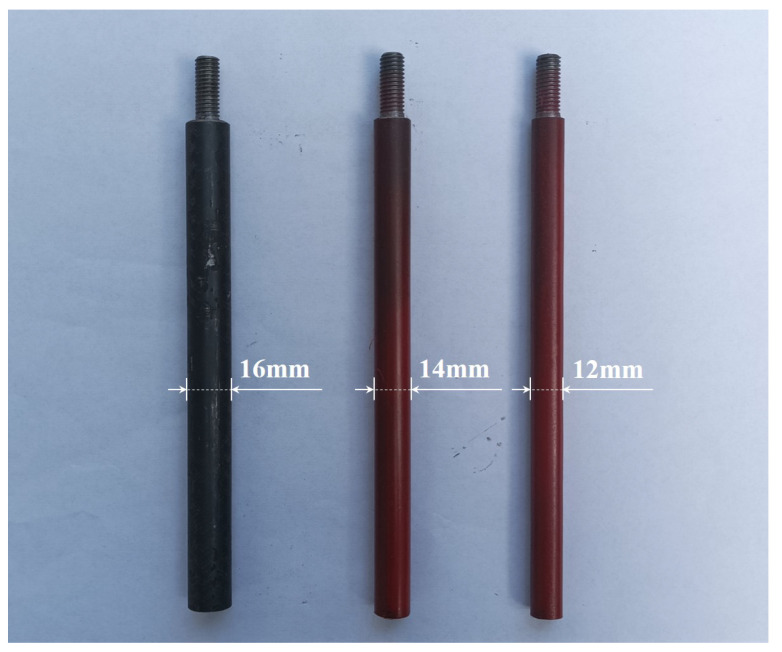
Three types of replacement columns.

During the long-term service of the structure, corrosion of the structural metal surface can lead to a decrease in the net cross-sectional area of the components, thereby reducing the load-bearing capacity. It can lead to serious effects on the structural safety. Therefore, the focus of this study was to accurately identify changes in the net cross-sectional area of structural components. In Figure 14, the structure is considered to be in a healthy state, with a net cross-sectional diameter of 16 mm for all columns. The experiment replaced the 16mm original column at the southeast corner of one or more floors in the structural model with columns with a cross-sectional diameter of 14 mm or 12 mm. The purpose was to simulate different degrees of corrosion damage. Table 7 summarizes the six damage scenarios simulated in this paper.

**Figure 15:**
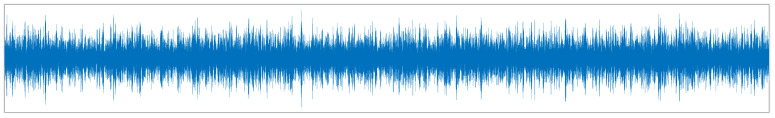
The acceleration response curves of the D.P.0 damage pattern in the south side on the first floor.

**Figure 16:**
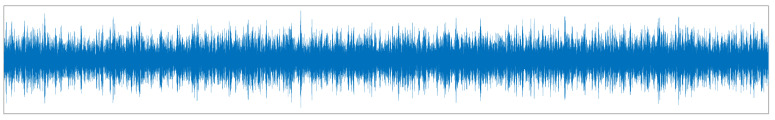
The acceleration response curves of the D.P.0 damage pattern in the north side on the first floor.

As shown in Figure 13, a controllable exciter (Donghua DH40100) with a stinger was used to apply a zero mean white Gaussian noise excitation to the first floor of the structure along the southeast–northwest diagonal. Four Donghua 1A401E acceleration sensors were installed at midspan positions on each of the four sides of every story slab to precisely measure the structural responses. A Donghua DH8303 data acquisition system was used to collect acceleration data. The acquisition rate was set at 250 Hz for all damage patterns, with each acquisition lasting 800 s and 200,000 data points being collected each time. Figure 15 and Figure 16 present the *y* direction acceleration response signals for the first floor in the undamaged state.

### 4.2. Comparative Analysis of Models in the Experimental Structure

To accurately assess the effectiveness and generalization capabilities of CGsformer, the acceleration response data for each direction on each floor were processed according to the data preprocessing method described in Section 3.1. Then, the dataset for each direction came out to 9372 samples. From these samples, 7500 samples were allocated for training the classifiers, and 1872 were set aside for testing after training. Finally, the preprocessed acceleration response time history data (4 floors × 2 translational directions) were used to train eight CGsformer classifiers.

In Table 8, the performance of the proposed CGsformer model is compared with the other four models, i.e., CNN, LSTM, Transformer, and Conformer. The CGsformer model demonstrated superior performance, thus achieving an average accuracy of 92.44%, which significantly outperformed the Conformer (91.71%), Transformer (87.60%), LSTM (83.92%), and CNN (78.43%) models. Noteworthy are the accuracies observed in the *y* direction of the first floor and the *x* direction of the second floor, where CGsformer reached accuracies of 93.91% and 92.97%, respectively. These outcomes are attributed to the model’s hierarchical learning approach. The CGsformer networks employs a multihead self-attention mechanism to capture the global information of signals, which helps to identify the periodic relationships of acceleration response signals. Meanwhile, it utilizes a convolution module to precisely capture the slight differences in acceleration response signals at short and adjacent moments. Furthermore, the graph convolution module embedded in the CGsformer enhances the model’s robustness against noise pollution. It does this by constructing a graph structure for global features and filtering out noise in global features through node learning, thereby enabling the convolution module to extract more effective local features. These innovative aspects ensure the efficacy and reliability of CGsformer in structural damage identification, with good generalization performance across various types of damages.

## 5. Conclusions

This paper presents an innovative deep learning model, called CGsformer, for detecting structural damages. The proposed CGsformer effectively extracts the global and local features of signals by employing a hierarchical learning approach from global to local. Additionally, the GCN is embedded after the multihead self-attention module for further propagating global features to adjacent nodes, which helps to better understand and express node features by incorporating local adjacency information. The proposed damage detection method based on the CGsformer was verified using simulation data from the IASC-ASCE benchmark structure and experimental data from a four-story, single-span, steel frame structure. Some valuable conclusions can be drawn from the validated results:

- The proposed damage detection model has demonstrated its feasibility in test setups with the IASC-ASCE simulated benchmark structure and a four-story, single-span, steel frame structure, thus achieving damage identification accuracies of 96.71% and 92.44%, respectively. These results not only validate the effectiveness of the CGsformer in identifying structural damage but also provide valuable insights for future research.
- The proposed CGsformer model exhibited high accuracy and robustness in limited datasets and noise-contaminated conditions. In the example of the IASC-ASCE benchmark structure, despite the noise level increasing from 0% to 50%, the detection accuracy only decreased by 1.81%. This means that the CGsformer can more effectively extract features from the acceleration response signal, thus showcasing strong noise resistance.

Although the proposed method achieved surprising performance through its learning manner from local to global, some limitations should also be noted. From an application perspective, the proposed method has not yet been tested in practical engineering. To this end, we plan to collaborate with industry partners to implement and validate our methods in real-world structural health monitoring scenarios. From a technical perspective, although the Transformer can achieve parallel computing compared to the RNN, it also increases the number of model parameters. Thus, we will consider compressing or pruning the model in the future work. Moreover, obtaining acceleration response data for structures with damage is a challenge. Thus, future research directions are intended to combine transfer learning with structural numerical simulation models for structure damage detection.

## Figures and Tables

**Table 1:** Normal pattern and three cases of damage patterns as defined in the IASC-ASCE SHM simulated benchmark structure.

Damage Pattern	Pattern Description
D.P.0	No damage.
D.P.1	All braces on the first floor have no stiffness.
D.P.2	All braces on the first and third floors have no stiffness.
D.P.4	One brace of the first floor and the third floor has no stiffness.

**Table 2:** The hyperparameter settings adopted in the CGsformer.

Setting	Value
Encoder Layers	4
Encoder Dim	512
Attention Heads	2
Conv Kernel Size	19
Multihead Attention Dropout	0.4
CGsformer Dropout	0.1

**Table 3:** Comparison results between CGsformer and other models.

Method	*ACC*	$F_{\mathrm{score}}$
CNN	0.8910	0.8903
LSTM	0.8822	0.8826
CNN-LSTM	0.9255	0.9258
Multihead CNN	0.9239	0.9238
Transformer	0.9407	0.9406
Conformer	0.9527	0.9528
**CGsformer**	**0.9671**	**0.9672**

**Table 4:** Statistical significance comparison of the proposed model with other models.

Model	ACC	ΔACC	CI	ΔCI
CNN	0.8910	0.0761	[0.8724, 0.9078]	[0.0562, 0.0960]
LSTM	0.8822	0.0849	[0.8630, 0.8996]	[0.0645, 0.1053]
CNN-LSTM	0.9255	0.0416	[0.9104, 0.9402]	[0.0240, 0.0592]
Multihead CNN	0.9239	0.0432	[0.9086, 0.9387]	[0.0255, 0.0609]
Transformer	0.9407	0.0264	[0.9261, 0.9532]	[0.0100, 0.0428]
Conformer	0.9527	0.0144	[0.9394, 0.9638]	[−0.0010, 0.0298]
CGsformer	0.9671	-	[0.9557, 0.9763]	-

**Table 5:** The effectiveness of GCN at various positions in the CGsformer.

Method	*ACC*	$F_{\mathrm{score}}$
Conformer	0.9527	0.9528
**CGsformer**	**0.9671**	**0.9672**
Attention Before	0.9631	0.9632
Convolution After	0.9623	0.9623

**Table 6:** Experimental results of 24 CGsformer classifiers (3 noise levels × 4 floors × 2 translational directions) using the acceleration response data collected from all acceleration sensors of the IASC-ASCE SHM Benchmark structure under 0%, 20%, and 50% noise levels.

Direction/Floor	Noise 0%	Noise 20%	Noise 50%
First Floor, *x* direction	0.9671	0.9607	0.9279
First Floor, *y* direction	0.9688	0.9712	0.9375
Second Floor, *x* direction	0.9776	0.9704	0.9583
Second Floor, *y* direction	0.9816	0.9752	0.9391
Third Floor, *x* direction	0.9824	0.9631	0.9383
Third Floor, *y* direction	0.9671	0.9535	0.9191
Fourth Floor, *x* direction	0.9575	0.9607	0.9543
Fourth Floor, *y* direction	0.9776	0.9832	0.9615
**Average**	**0.9725**	**0.9674**	**0.9544**

**Table 7:** Damage patterns as defined in the experimental structure model.

Damage Case	Description
D.P.0	Without damage (The columns at the southeast corner of floors 1–4 all have a diameter of 16 mm)
D.P.1	Replaced the column on the first floor with a 14 mm diameter column.
D.P.2	Replaced the column on the second floor with a 14 mm diameter column.
D.P.3	Replaced the column on the third floor with a 14 mm diameter column.
D.P.4	Replaced the column on the forth floor with a 14 mm diameter column.
D.P.5	Replaced the columns on the first and second floors with 14 mm and 12 mm diameter columns, respectively

**Table 8:** Identification accuracy results of the four stories and one-span steel frame structure from different models.

Floor/Direction	CNN	LSTM	Transformer	Conformer	CGsformer
First Floor, *x* direction	80.98	84.45	89.52	91.18	93.16
First Floor, *y* direction	77.78	82.10	87.01	93.37	93.91
Second Floor, *x* direction	76.01	86.59	86.43	91.93	92.97
Second Floor, *y* direction	82.05	84.93	87.23	93.48	93.56
Third Floor, *x* direction	81.20	83.60	84.61	88.67	90.03
Third Floor, *y* direction	76.33	86.37	85.95	91.72	92.25
Forth Floor, *x* direction	74.47	79.22	88.19	91.93	91.83
Forth Floor, *y* direction	78.63	84.13	91.82	91.40	92.20
**Average**	**78.43**	**83.92**	**87.60**	**91.71**	**92.44**

## Data Availability

The code for generating the datasets used in the numerical verification is available on the datacenterhub website at https://www.dropbox.com/sh/zpkqy5w371mnzam/AAA-Omuvwx72tjv5NhnhnPuMa?e=1&dl=0 (accessed on 3 July 2024). The datasets used in the experimental verification are available upon request from the corresponding author. The datasets are not available to the public, as they are the preliminary results of an ongoing research project carried out in collaboration. Furthermore, this information will be used in future technological developments and will be subject to intellectual property protection.
